# The risk of death associated with delayed coronary artery bypass surgery

**DOI:** 10.1186/1472-6963-6-85

**Published:** 2006-07-05

**Authors:** Boris G Sobolev, Adrian R Levy, Lisa Kuramoto, Robert Hayden, James M Brophy, J Mark FitzGerald

**Affiliations:** 1 Department of Health Care and Epidemiology, University of British Columbia, Vancouver, Canada; 2 Centre for Clinical Epidemiology and Evaluation, Vancouver Coastal Health Research Institute, Vancouver, Canada; 3 Centre for Health Evaluation and Outcome Sciences, St. Paul's Hospital, Vancouver, Canada; 4 Department of Surgery, Royal Columbian Hospital, New Westminster, Canada; 5 McGill University Health Centre, Montreal, Canada

## Abstract

**Background:**

The detrimental effect of delaying surgical revascularization has been estimated in randomized trials and observational studies. It has been argued that the Kaplan-Meier method used in quantifying the hazard of delayed treatment is not appropriate for summarizing the probability of competing outcomes. Therefore, we sought to improve the estimates of the risk of death associated with delayed surgical treatment of coronary artery disease.

**Methods:**

Population-based prospective study of 8,325 patients registered to undergo first time isolated coronary artery bypass grafting (CABG) in any of the four tertiary hospitals that provide cardiac care to adult residents of British Columbia, Canada. The cumulative incidence of pre-operative death, the cumulative incidence of surgery, and the probability that a patient, who may die or undergo surgery, dies if not   operated by certain times over the 52-week period after the decision for CABG were estimated. The risks were quantified separately in two groups: high-severity at presentation were patients with either persistent unstable angina or stable angina and extensive coronary artery disease, and low-severity at presentation were stable symptomatic patients with limited disease.

**Results:**

The median waiting time for surgery was 10 weeks (interquartile range [IQR] 15 weeks) in the high-severity group and 21 weeks (IQR 30 weeks) in the low-severity group. One percent of patients died before surgery:   54 in the high-severity and 26 in the low-severity group. For 58 (72.5%) patients, death was related to CVD (acute coronary syndrome, 33; chronic CVD, 16; other CVD, 4; and sudden deaths, 5). The overall death rate from all causes was 0.61 (95% CI 0.48-0.74) per 1,000 patient-weeks, varying from 0.62 (95% CI 0.45-0.78) in the high-severity group to 0.59 (95% CI 0.37-0.82) in the low-severity group. After adjustment for age, sex, and comorbidity, the all-cause death rate in the low-severity group was similar to the high-severity group (OR = 1.02, 95% CI 0.64-1.62). The conditional probability of death was greater in the high-severity   group than in the low-severity group both for all-cause mortality (p = 0.002) and cardiovascular deaths (p <0.001).

**Conclusion:**

The probability of death conditional on not having undergone a required CABG increases with time spent on wait lists.

## Background

Coronary-artery bypass grafting (CABG) is indicated for revascularizing patients who have limiting angina that persists despite optimal medical treatment and suitable coronary anatomy. In a meta-analysis of seven randomized controlled trials of immediate CABG versus medical therapy, surgery has been shown to improve prognosis in stable symptomatic patients with left main coronary disease, triple vessel disease, or two vessel disease involving a significant stenosis of the proximal left anterior descending coronary artery [1]. It has been argued that some of the survival benefits could be lost due to additional deaths resulting from a longer wait for required revascularization [2]. Methodologically, not including pre-operative deaths implies that survival time begins at procedure; and, therefore, treatment effect is implicitly conditioned on surviving to treatment [3]. Also, when reporting mortality among patients who may die or undergo surgery special statistical techniques must be used to isolate the effect of competing risks of surgery and death [5]. The Kaplan-Meier method used in reports on these trials is not appropriate for describing the probability of competing outcomes over time [4].

The detrimental effect of delaying CABG surgery has been estimated as well in observational studies of patients whose treatment was delayed due to a rationing of access to care. Population-based studies show that from 0.4 to 1.3 percent of patients scheduled for CABG die preoperatively [6-8]. Again, these proportions cannot be studied by the Kaplan-Meier method as they are affected by the incidence of both surgery and death. Therefore, it is not clear whether the low observed probabilities of death indicate a true low risk of death or appropriate timing of surgery.

One measure suggested for summarizing the risk of death over time in competing-risk setting is the probability of death conditional on not having experienced the competing event by a certain time [9,10]. Using this approach, we sought to improve the estimates of the risk of death associated with delayed coronary artery bypass surgery in patients requiring and suitable for surgical revascularization. We, therefore, estimated the time-dependent probability of death, given that CABG was not performed by certain times, using data from a prospective database of all adult patients who were accepted for isolated first time coronary artery surgery in British Columbia [BC], Canada.

## Methods

The University of British Columbia Ethics Board approved the study protocol.

### Data sources

A population-based cardiac registry contains the time of registration on wait lists for CABG and the time of procedure, or removal from wait lists without surgery, for all patients who have been accepted for surgical coronary revascularization in any of the four tertiary hospitals that provide cardiac care to adult residents of BC since 1991 [11]. The reliability of demographic and clinical data in the registry has been described elsewhere [12].

The date and cause of death for the registry records were obtained from BC Linked Health Database Deaths File for 1990 through 2001 [13]. Causes of death were coded according to the International Classification of Diseases, 9th revision (ICD-9) [14]. Cardiovascular deaths were all those with ICD-9 codes 410–439 plus sudden deaths due to unknown causes. Data on coexisting medical conditions were retrieved from the BC Linked Health Database Hospital Separations File using diagnoses reported in discharge abstracts created during the calendar year before registration for CABG [15].

### Patients

We studied records of patients for whom surgical revascularization was indicated at the time of consultation with a cardiac surgeon. For this analysis, patients were divided into high-severity and low-severity at presentation groups according to angiographic findings, symptom severity and left ventricular dysfunction (ejection fraction less than 50%) [16].

The high-severity group consisted of patients with either persistent unstable angina or stable angina and extensive CAD (left-main stenosis more than 50%, triple-vessel disease, or double-vessel disease with significant proximal left anterior descending stenosis and impaired left ventricular function). The low-severity group consisted of stable symptomatic patients with limited CAD (double-vessel disease with no lesion in the proximal left anterior descending artery and normal left ventricular function or single-vessel disease with significant proximal left anterior descending stenosis).

There were 8,494 patients identified who required isolated (did not include a valve replacement procedure) first time coronary artery bypass surgery in these two groups between January 1991 and December 2000. We excluded 169 records of the patients who were removed on the registration date (50), had missing operating room reports (4), or had immediate access to surgery (115). Of those, 161 eventually underwent surgery; seven died; 75 became unfit for surgery; 100 declined surgery; 16 were transferred to another surgeon or hospital; and 96 were removed from wait lists for other reasons. The baseline characteristics of patients are shown in Table 1. The remaining 8,325 patients had either a surgery date or a date and reason for removal without surgery. The study period ended in December 2001, allowing only 52 weeks of follow-up after the last patients were added to the list. Therefore, we restricted the analysis to the first 52 weeks after registration so that 455 (5.5%) patients remaining on the lists at 12 months were censored. 

### Statistical analysis

The primary outcomes were the occurrence of death from all causes and death related to cardiovascular disease (CVD) while awaiting coronary artery surgery. The date of the operating room booking request from surgeon served as the date of decision for surgery and registration on a wait list.

In the participating hospitals, surgical wait lists are used to hold patient names until the surgery can be scheduled. Patients are also removed from the wait lists without surgery if they die, reconsider the decision to undergo surgery, accept surgery from another surgeon, move out of the province, or if their conditions deteriorate so that surgery is no longer possible. Details regarding the wait-list management were published elsewhere [11].

The risk of death as a function of treatment delay is described by the probability that a patient dies conditional on not having undergone surgery by a certain time after the registration on a wait list for CABG. The conditional probability function (CPF) of death is interpreted as the cumulative incidence of deaths by a certain wait-list week among cardiac surgery patients who had not yet undergone CABG by that time.

To estimate CPF of death, we first estimated separately the cumulative incidence of death and the cumulative incidence of surgery over time while treating wait-list removals, other than surgery and death, as censored observations. The cumulative incidence function of an event is defined as the integration over time of the product of the event rate and the probability of remaining on the list [17]. The following section describes the estimation procedure.

Suppose the events of death and surgery occur at *E *distinct, unevenly spaced, ordered times, *t*_*i*_, for *i *= 1,2,..., *E*, and define *t*_0 _= 0. Using Gooley's notation [5], let *e*_*i *_be the number of deaths at time *t*_*i*_, *r*_*i *_be the number of surgeries at time *t*_*i*_, *c*_*i *_be the number of censored events at time *t*_*i*_, and *n*_*i *_= *n*_*i*-1 _- (*e*_*i *_+ *r*_*i *_+ *c*_*i*_) be the number of patients still waiting beyond time *t*_*i*_, where *n*_0 _is the initial number of patients at risk. As described by Kalbfleisch and Prentice, a non-parametric estimator for the cumulative incidence of death at time *t*, *F*_1_(*t*), is given by the following formula [18]:

F^1(t)=∑{i|ti≤t}eini−1∏{j|tj≤ti−1}(1−ej+rjnj−1).     (1)
 MathType@MTEF@5@5@+=feaafiart1ev1aaatCvAUfKttLearuWrP9MDH5MBPbIqV92AaeXatLxBI9gBaebbnrfifHhDYfgasaacH8akY=wiFfYdH8Gipec8Eeeu0xXdbba9frFj0=OqFfea0dXdd9vqai=hGuQ8kuc9pgc9s8qqaq=dirpe0xb9q8qiLsFr0=vr0=vr0dc8meaabaqaciaacaGaaeqabaqabeGadaaakeaacuWGgbGrgaqcamaaBaaaleaacqaIXaqmaeqaaOGaeiikaGIaemiDaqNaeiykaKIaeyypa0ZaaabuaeaadaWcaaqaaiabdwgaLnaaBaaaleaacqWGPbqAaeqaaaGcbaGaemOBa42aaSbaaSqaaiabdMgaPjabgkHiTiabigdaXaqabaaaaaqaaiabcUha7jabdMgaPjabcYha8jabdsha0naaBaaameaacqWGPbqAaeqaaSGaeyizImQaemiDaqNaeiyFa0habeqdcqGHris5aOWaaebuaeaadaqadiqaaiabigdaXiabgkHiTmaalaaabaGaemyzau2aaSbaaSqaaiabdQgaQbqabaGccqGHRaWkcqWGYbGCdaWgaaWcbaGaemOAaOgabeaaaOqaaiabd6gaUnaaBaaaleaacqWGQbGAcqGHsislcqaIXaqmaeqaaaaaaOGaayjkaiaawMcaaaWcbaGaei4EaSNaemOAaOMaeiiFaWNaemiDaq3aaSbaaWqaaiabdQgaQbqabaWccqGHKjYOcqWG0baDdaWgaaadbaGaemyAaKMaeyOeI0IaeGymaedabeaaliabc2ha9bqab0Gaey4dIunakiabc6caUiaaxMaacaWLjaWaaeWaceaacqaIXaqmaiaawIcacaGLPaaaaaa@6E51@

Similarly, a non-parametric estimator for the cumulative incidence of surgery at time *t*, *F*_2_(*t*), is

F^2(t)=∑{i|ti≤t}rini−1∏{j|tj≤ti−1}(1−ej+rjnj−1).     (2)
 MathType@MTEF@5@5@+=feaafiart1ev1aaatCvAUfKttLearuWrP9MDH5MBPbIqV92AaeXatLxBI9gBaebbnrfifHhDYfgasaacH8akY=wiFfYdH8Gipec8Eeeu0xXdbba9frFj0=OqFfea0dXdd9vqai=hGuQ8kuc9pgc9s8qqaq=dirpe0xb9q8qiLsFr0=vr0=vr0dc8meaabaqaciaacaGaaeqabaqabeGadaaakeaacuWGgbGrgaqcamaaBaaaleaacqaIYaGmaeqaaOGaeiikaGIaemiDaqNaeiykaKIaeyypa0ZaaabuaeaadaWcaaqaaiabdkhaYnaaBaaaleaacqWGPbqAaeqaaaGcbaGaemOBa42aaSbaaSqaaiabdMgaPjabgkHiTiabigdaXaqabaaaaaqaaiabcUha7jabdMgaPjabcYha8jabdsha0naaBaaameaacqWGPbqAaeqaaSGaeyizImQaemiDaqNaeiyFa0habeqdcqGHris5aOWaaebuaeaadaqadiqaaiabigdaXiabgkHiTmaalaaabaGaemyzau2aaSbaaSqaaiabdQgaQbqabaGccqGHRaWkcqWGYbGCdaWgaaWcbaGaemOAaOgabeaaaOqaaiabd6gaUnaaBaaaleaacqWGQbGAcqGHsislcqaIXaqmaeqaaaaaaOGaayjkaiaawMcaaaWcbaGaei4EaSNaemOAaOMaeiiFaWNaemiDaq3aaSbaaWqaaiabdQgaQbqabaWccqGHKjYOcqWG0baDdaWgaaadbaGaemyAaKMaeyOeI0IaeGymaedabeaaliabc2ha9bqab0Gaey4dIunakiabc6caUiaaxMaacaWLjaWaaeWaceaacqaIYaGmaiaawIcacaGLPaaaaaa@6E6F@

The formula for a Taylor series approximation of the variance for the cumulative incidence of an event was developed by Gaynor [19].

The CPF of death, among those who remained untreated, is defined as the ratio of the cumulative incidence of death and the complement of cumulative incidence of surgery [10]. We used the non-parametric estimator for the calculation of the CPF of death [9]:

CP^1(t)=F^1(t)1−F^2(t).     (3)
 MathType@MTEF@5@5@+=feaafiart1ev1aaatCvAUfKttLearuWrP9MDH5MBPbIqV92AaeXatLxBI9gBaebbnrfifHhDYfgasaacH8akY=wiFfYdH8Gipec8Eeeu0xXdbba9frFj0=OqFfea0dXdd9vqai=hGuQ8kuc9pgc9s8qqaq=dirpe0xb9q8qiLsFr0=vr0=vr0dc8meaabaqaciaacaGaaeqabaqabeGadaaakeaadaqiaaqaaiabdoeadjabdcfaqbGaayPadaWaaSbaaSqaaiabigdaXaqabaGccqGGOaakcqWG0baDcqGGPaqkcqGH9aqpdaWcaaqaaiqbdAeagzaajaWaaSbaaSqaaiabigdaXaqabaGccqGGOaakcqWG0baDcqGGPaqkaeaacqaIXaqmcqGHsislcuWGgbGrgaqcamaaBaaaleaacqaIYaGmaeqaaOGaeiikaGIaemiDaqNaeiykaKcaaiabc6caUiaaxMaacaWLjaWaaeWaceaacqaIZaWmaiaawIcacaGLPaaaaaa@4667@

Its variance was determined by Pepe in [9]. To estimate the cumulative incidence of events, CPF of death, and the corresponding confidence intervals, we used Matlab version 7.0.1 [see Additional file1]. A two-sample test was used to compare the CPF between the study groups [9]. The cumulative incidence of surgery was compared between the two groups by the Gray's test [20].

We used discrete-time survival regression models to evaluate the combined effect of clinical factors that identify the patient groups in this study on the death rate, while adjusting for age, sex, and comorbidity [21]. The likelihood ratio test was used to assess whether the models were consistent with the data [22]. Existing literature suggest that age, sex and comorbidities may be potential confounders. Elderly patients are more likely to undergo revascularization as an urgent procedure. The smaller coronary vessel diameters may account for higher risk of adverse events in women. Co-existing medical conditions may delay scheduling surgery. All of these factors were entered into the regression models. In particular, each patient was classified as 1) presenting with congestive heart failure, diabetes, chronic obstructive pulmonary disease, cancer or rheumatoid arthritis, 2) presenting with other co-existing chronic conditions as defined in [23], or 3) presenting with no co-existing conditions.

## Results

At 52 weeks of follow-up, 7,155 (85.9%) patients underwent surgery, 80 (1.0%) died while awaiting surgery, 455 (5.5%) patients were remaining on the lists, and 635 (7.6%) dropped out during follow-up for various reasons: became unfit to surgery (166), declined surgery (181), transferred to another surgeon or hospital (93), received other surgery (21), or removed from the list due to other reasons (174), Table 2. Over 10% of low-severity patients and less than 5% of high-severity patients were still untreated at 52 weeks.

**Figure 1:**
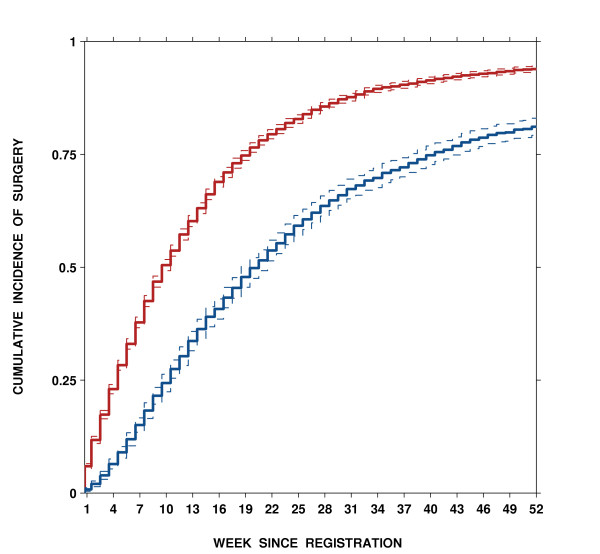
Estimated cumulative incidence of surgery and 95% CIs by week since registration in high-severity (red) and low-severity (blue) groups; two-sample test = 411.8, *p *< 0.001.

The extent of disease was a major factor influencing time to surgery. The median waiting time for surgery was 10 weeks (interquartile range [IQR] 15 weeks) in the high-severity group and 21 weeks (IQR 30 weeks) in the low-severity group. The differences in the cumulative incidence of surgery were significant over time between groups with higher incidence in the high-severity group, (Gray's two-sample test = 411.8, *p *< 0.001), Figure 1.

A total of 5,722 surgeries over 87,674 patient-weeks in the high-severity and 1,433 surgeries over 43,817 patient-weeks in the low-severity group were done. The average surgery rate was 6.5 per 100 patients per week of delay in the high-severity group compared to 3.3 in the low-severity, the odds ratio (OR) = 0.50 (95% confidence interval [CI] 0.47–0.53), after adjustment for age, sex, and comorbidity. The log-likelihood ratio test, 619.9, df = 5, *p *< 0.001, does not support the global null hypothesis, suggesting that the model is consistent with data.

One percent of patients died before surgery: 54 in the high-severity and 26 in the low-severity group. For 58 (72.5%) patients, death was related to CVD (acute coronary syndrome, 33; chronic CVD, 16; other CVD, 4; and sudden deaths, 5).

The overall death rate from all causes was 0.61 (95% CI 0.48–0.74) per 1,000 patient-weeks, varying from 0.62 (95% CI 0.45–0.78) in the high-severity group to 0.59 (95% CI 0.37–0.82) in the low-severity group. After adjustment for age, sex, and comorbidity, the all-cause death rate in the low-severity group was similar to the high-severity group (OR = 1.02, 95% CI 0.64–1.62). The log-likelihood ratio, 16.2, df = 5, *p *< 0.01, test does not support the global null hypothesis, suggesting that, at least one regression coefficient differs from zero, and therefore, the model is consistent with data.

**Figure 2:**
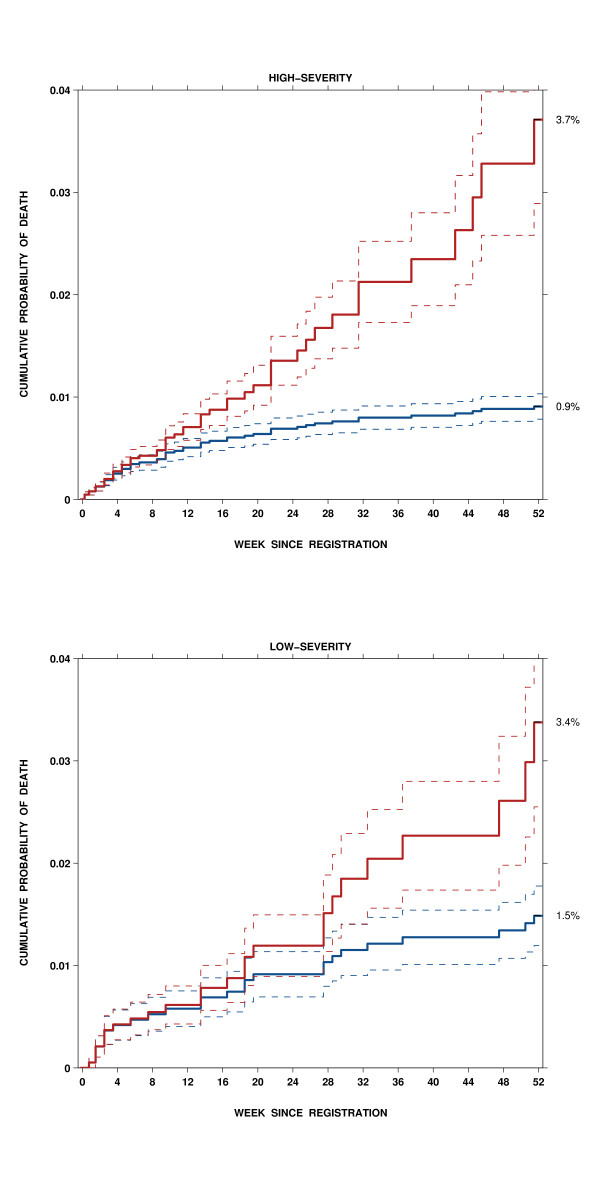
Estimated probabilities of preoperative death, as cumulative incidence function (blue) and Kaplan-Meier (red) and their standard errors, by week since registration in high-severity and low-severity group.

Figure 2 shows the relationship between wait time and the probability of preoperative death from all causes by severity group, which was estimated by non-parametric methods as described in a previous section and also by the Kapaln-Meier method. Although the all-cause death rate is similar for the two groups, 0.62 versus 0.59 per 1,000 patient-weeks, longer wait times contributed to a higher cumulative incidence of death in the low-severity than high-severity group [11]. The non-parametric cumulative incidence function provides lower probabilities of death than the Kaplan-Meier method [24]. At 52 weeks since registration, the Kaplan-Meier estimates are about 4 times greater (3.7% versus 0.9%) in the high-severity group, and 2 times greater (3.4% versus 1.5%) in the low-severity group.

**Figure 3:**
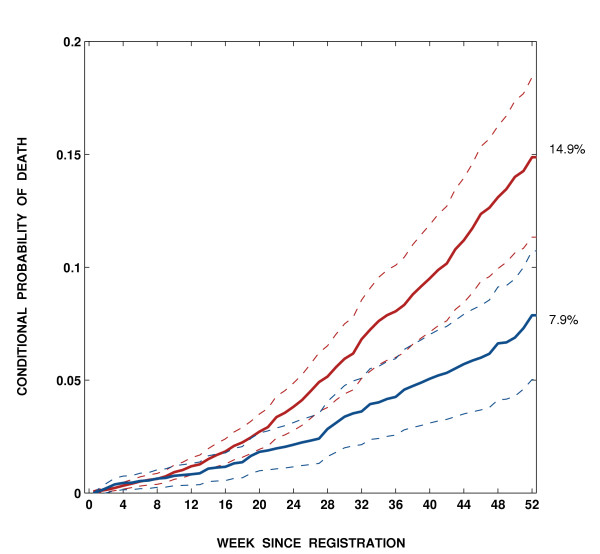
Estimated conditional probability for all-cause death and 95% confidence intervals, by week since registration in high-severity (red) and low-severity groups (blue); two-sample test = 3.1, *p *= 0.002.

To compare proportions of patients dying by a certain time among those who had not undergone surgery by that time, we calculated the conditional probability of death in each group. The non-parametric estimate of the conditional probability of death was derived from the ratio of the cumulative incidence of death and the complement of the cumulative incidence of surgery. The conditional probability for death from all causes was greater in the high-severity group than in the low-severity group (Pepe's two-sample test = 2.8, *p *= 0.002), Figure 3. Among patients who had not undergone CABG by 8, 16, 32 and 52 weeks, the probability to die from all causes was 0.6% (standard error [SE] 0.1), 1.8% (0.3), 6.8% (0.9) and 14.9% (1.8) in the high-severity group, and 0.6% (0.2), 1.2% (0.3), 3.6% (0.8) and 7.9% (1.5) in the low-severity group.

**Figure 4:**
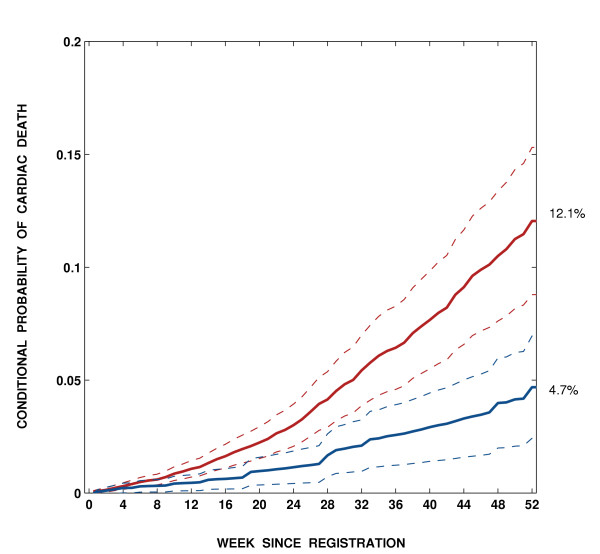
Estimated conditional probability for cardio-vascular death and 95% confidence intervals, by week since registration in high-severity (red) and low-severity (blue) groups; two-sample test = 3.6, *p *< 0.001.

The conditional probability for CVD death was greater in the high-severity group than in the low-severity group (Pepe's two-sample test = 3.6, *p *= 0.0002), Figure 4. Among patients who had not undergone CABG by 8, 16, 32 and 52 weeks, the probability to die from CVD was 0.6% (0.1), 1.6% (0.3), 5.4% (0.8) and 12.1% (1.7) in the high-severity group, and 0.3% (0.1), 0.6% (0.2), 2.1% (0.6) and 4.7% (1.2) in the low-severity group.

## Discussion

In this prospective study of 8,325 consecutive patients we have reported outcomes of delaying coronary artery surgery in two groups of patients for whom isolated CABG was indicated. The high-severity group included patients with either persistent unstable angina or stable angina and extensive CAD at presentation. The low-severity group included stable symptomatic patients with limited CAD.

In each group, we have estimated the time-dependent conditional probability that a patient, who may die or undergo surgery, dies if not operated by certain times. These probabilities, which summarize competing risks data on wait-lists, have not been previously reported for untreated CAD patients for whom CABG was indicated. As noted by Pepe [9], the conditional probability function is not a predicted probability and its interpretation does not require any implicit assumptions. It is simply the proportion of patients who have died among those who remained untreated. We found that among patients delayed without treatment for 52 weeks, an estimated 14.9% die in the high-severity group and 7.9% die in the low-severity group from all causes. Similarly, an estimated 12.1% and 4.7% die from CVD in these two groups.

We report on all-cause and CVD mortality because the accuracy of death certificate codes is a concern in this analysis [25], whereas using all-cause mortality could not have induced any bias in the results. Some evidence that coding of CVD deaths is accurate comes from a Canadian study in which the false positive rate was 2.1% and the false negative rate was 0.4% for myocardial infarction coded as an underlying cause of death [26]. We also argue that there is no reason to suspect that there would be differential coding of death certificates according to urgency of treatment as the physician completing the death certificate would not necessarily have been aware of the assigned urgency at registration for CABG.

In quantifying the risk of preoperative death among patients needing CABG, the Kaplan-Meier method is commonly used to estimate the cumulative probability of death by certain times after registration for the operation [27-29]. It has been established, however, the Kaplan-Meier method is not appropriate for describing the probabilities of competing events since its complement overestimates the proportion of events [4]. This method produces valid probability estimates only in a hypothetical situation where all competing risks can be removed without altering the risk of death.

Other investigators have reported the incidence of preoperative death per time unit of waiting for CABG [6-8,27,29-31]. Although accurately describing the instantaneous hazard, death rates can not be converted into probabilities of death without an unrealistic and unverifiable assumption that time to surgery and time to death are independent [5]. Plomp and colleagues have reported on the variation in time to deaths among those who died before surgery [32], but the proportion of CABG candidates dying over follow-up could not be derived from their figures.

Methodologically, measuring risk of death as a function of treatment delay in patients awaiting the treatment is similar to quantifying the risk of death during follow-up in a population exposed to competing events [4]. Therefore, an alternative approach to summarize competing risks data is to estimate the proportion of patients dying by a certain time among those not receiving treatment by that time [10]. We used the conditional probability function suggested as a method for summarizing multiple endpoints in the competing-risks setting by Pepe [33].

There were important limitations to our study. First, we have not adjusted the CPF of death for available covariates as the statistical methodology for that is yet to be developed. Therefore, there is a concern that some other factors can confound the difference between the two study groups. However, in our data set both study groups were distributed similarly over age, sex and comorbidity category and differed only by the extent of CAD. The number of deaths observed does not permit the analysis across strata. A new update of data in the future will perhaps allow us to report CPFs by important covariates, such as sex and age. Also, our analysis lacks data on socioeconomic status. Given social class differences in access to healthcare and mortality, socioeconomic status is a potential confounding factor for the observed association between time to CABG and the risk of death [34]. One important issue is preferential allocation of hospital resources [35]. It remains unclear whether directly admitting patients of low priority is done to circumvent long wait lists, or to substitute for cancelations on the operating room schedule [11].

The quality of information on dates of registration and removal is a concern in this analysis as well. Although we considered the date of the booking request as the date of decision for surgery, no audit was conducted to verify the accuracy of coding dates in BCCR records.

In conclusion, the contribution of this paper is the estimated conditional probabilities of death in relation to different delays in the treatment of patients requiring and suitable for CABG. These summary probabilities derived from the population-based prospective database suggest that the risk of death among those remaining untreated increases with time on wait lists.

## Conclusion

Our findings have implications for policies related to access to elective cardiac surgery. First, in deciding on the duration of time that the treatment of elective patients can be safely delayed, surgeons and policy makers should be aware that the probability of death among untreated patients does not remain constant over time. Second, implicit in priority wait lists is the perception of a low risk of pre-operative death in less severe patients. Our results demonstrate that policy makers should be aware of an 8% risk of death in untreated patients with coronary artery disease judged to be low-severity at presentation, if there is a protracted delay before revascularization.

## Competing interests

The author(s) declare that they have no competing interests.

## Authors' contributions

BS conceived the study concept and design, participated in analysis and interpretation, and drafted the manuscript. AL participated in data acquisition and critically revised the manuscript. LK performed statistical analysis and drafted the manuscript. RH participated in data acquisition. JB critically revised the manuscript. JMF critically revised the manuscript and has been involved in drafting the manuscript. All authors read and approved the final manuscript.

## Pre-publication history

The pre-publication history for this paper can be accessed here:



## Supplementary Material

Additional File 1 Appendix Matlab programs for non-parametric estimators of cumulative incidence and conditional probability functions Click here for file

## Figures and Tables

**Table 1:** Characteristics of 8,325 patients (6,405 in high-severity and 1,920 in low-severity) registered for isolated coronary artery bypass surgery in British Columbia, 1991–2000

**Characteristic**	**All patients N (%)**	**High-severity N (%)**	**Low-severity N (%)**
**Age group (y)**			

<50	679 (8.2)	496 (7.7)	183 (9.5)
50–59	1841 (22.1)	1397 (21.8)	444 (23.1)
60–69	3167 (38.0)	2457 (38.4)	710 (37.0)
70–79	2478 (29.8)	1933 (30.2)	545 (28.4)
≥80	160 (1.9)	122 (1.9)	38 (2.0)

**Sex**			

Women	1473 (17.7)	1102 (17.2)	371 (19.3)
Men	6852 (82.3)	5303 (82.8)	1549 (80.7)

**Comorbidity at registration**			

Major conditions*	1775 (21.3)	1358 (21.2)	417 (21.7)
Other conditions ^†^	2137 (25.7)	1723 (26.9)	414 (21.6)
None	4413 (53.0)	3324 (51.9)	1089 (56.7)

**Coronary anatomy**			

Left-main stenosis	990 (11.9)	990 (15.5)	0 (0.0)
Multi- vessel disease^‡^	6672 (80.1)	4986 (77.8)	1686 (87.8)
Limited disease^§^	663 (8.0)	429 (6.7)	234 (12.2)

*congestive heart failure, diabetes, chronic obstructive pulmonary disease, rheumatoid arthritis, cancer.

^†^peripheral vascular disease, cerebrovascular disease, dementia, peptic ulcer disease, hemiplegia, renal disease, or liver disease.

^‡^3 or 2-vessel disease with PLAD.

^§^2-vessel disease with no PLAD or 1-vessel disease with PLAD

**Table 2:** Number of patients (%) by 52-week outcome of registration for isolated coronary artery bypass surgery

**Outcomes**	**All patients N(%)**	**High-severity N(%)**	**Low-severity N(%)**
**Underwent surgery**	7155 (85.9)	5722 (89.3)	1433 (74.6)

**Removed without surgery**			

Died while waiting	80 (1.0)	54 (0.8)	26 (1.4)
Became unfit for surgery	166 (2.0)	90 (1.4)	76 (4.0)
Patient request	181 (2.2)	131 (2.0)	50 (2.6)
Transferred or moved	93 (1.1)	62 (1.0)	31 (1.6)
Other surgery	21 (0.3)	13 (0.2)	8 (0.4)
Other reason	174 (2.1)	117 (1.8)	57 (3.0)

**Still on wait list**	455 (5.5)	216 (3.4)	239 (12.4)
