# Supplementary material for: The risk of death associated with delayed coronary artery bypass surgery
Source: BMC Health Serv Res. 2006 Jul 5;6:85. doi: 10.1186/1472-6963-6-85 (PMC1574305; doi:10.1186/1472-6963-6-85)
Supplement: Additional File 1 — Appendix Matlab programs for non-parametric estimators of cumulative incidence and conditional probability functions [file 1472-6963-6-85-S1.pdf]

Appendix

Matlab programs for non-parametric estimators of cumulative incidence and conditional probability functions.

```
%% ‘getcif’ is a function to compute the cumulative incidence for event of interest
%%
%% Input:
%%      N, total number at risk (integer)
%%      e, number of events of interest at current time point (vector of integers)
%%      r, number of competing events at current time point (vector of integers)
%%      c, number of censored observations at current time point (vector of integers)
%%      t, distinct, ordered times (vector of integers/doubles)
%%
%%      Note that the elements of ‘e’, ‘r’, and ‘c’ should correspond to the same time points
%%
%% Output:
%%      F, estimated cumulative incidence function for event of interest at each time point
%%        in ‘t’ (vector of doubles)
%%      varF, estimate variance of cumulative incidence function for event of interest at each
%%        time point in ‘t’
%%      t, distinct, ordered times (vector of integers/doubles)
%%
%% Developed by Lisa Kuramoto (2004/08/18); Reviewed by Boris Sobolev (2004/09/01); Revised by Lisa
%% Kuramoto (2005/05/20).
%%
%% Based on Kalbfleisch and Prentice (1980) and Gaynor (1993).

function [F, varF, t] = getcif(N,e,r,c,t)

%% estimate cumulative incidence function %%

% number of distinct, ordered time points
ntme=length(e);

% number at risk beyond time point
n=N-cumsum(sum([e r c],2));

% number at risk at time point
Y=[N; n(1:(ntme-1))];

% total number of events at time point
dd=e+r;

% hazard of event of interest
lam=e./Y;

% overall survival
pS=1-dd./Y;
pS=[1; pS];
pS=pS(1:(length(pS)-1));
S=cumprod(pS);
```

```
% cumulative incidence for event of interest
F=cumsum(lam.*S);


%% estimate variance of cumulative incidence function %%

% estimated survival function
tmp2=[1; cumprod(1+(c(1:(ntme-1)))./n(1:(ntme-1)))];
KMc=cumsum(dd.*tmp2)/N;
if any(isnan(KMc))
   indx=ntme-sum(isnan(KMc));
   KMc(isnan(KMc))=KMc(indx);
end;
S=1-KMc;
S=[1; S];
S=S(1:(length(S)-1));

% covariance matrix of lamS
ny=length(Y)-1;
tmp=dd(1:ny)./(Y(1:ny).*(Y(1:ny)-dd(1:ny)));
sumtmp=cumsum(tmp);
sumtmp=[0; sumtmp];

lamS=lam.*S;

vlamS=zeros(ntme,1);
ind=(e~=0);
vlamS(ind)=lamS(ind).^2.*( (Y(ind)-e(ind))./(e(ind).*Y(ind)) + sumtmp(ind) );

covmtx=zeros(ntme,ntme);
tmpmtx=zeros(ntme,ntme);
for i=1:(ntme-1),
   for j=(i+1):ntme,
      ind=(e(i)~=0 & e(j)~=0);
      covmtx(i,j)=ind*lamS(i)*lamS(j)*(-1/Y(i)+sumtmp(i));
      covmtx(j,i)=covmtx(i,j);
      tmpmtx(i,j)=covmtx(i,j);
   end;
   covmtx(i,i)=vlamS(i);
end;
covmtx(ntme,ntme)=vlamS(ntme);


% compute variance of F
crstrm=sum(tmpmtx,1)';
varF=cumsum(vlamS)+2*cumsum(crstrm);
```

```
%% ‘getcp’ is a function to compute the conditional probability function for event of interest
%%
%% Input:
%%      N, total number at risk (integer)
%%      e, number of events of interest at current time point (vector of integers)
%%      r, number of competing events at current time point (vector of integers)
%%      c, number of censored observations at current time point (vector of integers)
%%      t, distinct, ordered times (vector of integers/doubles)
%%
%%      Note that the elements of ‘e’, ‘r’, and ‘c’ should correspond to the same time points
%%      in ‘t’.
%%
%% Output:
%%      CP, estimated conditional probability function for event of interest at each time point
%%        in ‘t’ (vector of doubles)
%%      varCP, estimated variance of conditional probability function for event of interest at
%%        each time point in ‘t’ (vector of doubles)
%%      t, distinct, ordered times (vector of integers/doubles)
%%
%% Helper function:
%%     ‘getcif’ to compute the cumulative incidence function
%%
%% Developed by Lisa Kuramoto (2004/08/13); Reviewed by Boris Sobolev (2004/08/18); Revised by Lisa
%% Kuramoto (2004/08/27).
%%
%% Based on Pepe (1993).

function [CP, varCP, t] = getcp(N,e,r,c,t)

%% estimate conditional probability function %%

% number of distinct, ordered time points
ntme=length(e);

% cumulative incidence for event of interest
[F1 varF1 t1] = getcif(N,e,r,c,t);

% cumulative incidence for competing event
[F2 varF2 t2] = getcif(N,r,e,c,t);

% conditional probabilities for event of interest
CP=F1./(1-F2);

%% estimate variance for conditional probability function %%

% number at risk at time point
Y=[N; n(1:(ntme-1))];

% overall survival
[Fo varFo to] = getcif(N,e+r,zeros(length(e),1),c,t);
S=1-Fo;
```

```
% variance of CP
trm1=S.^2./(1-F2).^4;
num=(1-F2).^2.*e + F1.^2.*r;
den=Y.*(Y-1);
smd=num./den;
cs=cumsum(smd);

varCP=trm1.*cs;
```
